# Immunogenicity of a single dose of the 17DD yellow fever vaccine in a cohort of adults and children in a non-endemic area, and its association with dengue and Zika seropositivity

**DOI:** 10.1371/journal.pntd.0012993

**Published:** 2025-04-09

**Authors:** Camylla Veloso Valença Saucha, Maria de Lourdes de Sousa Maia, Eduardo Sérgio Soares Sousa, Patrícia Mouta Nunes de Oliveira, Janaína Reis Xavier, Thalita da Matta de Castro, Robson Leite de Souza Cruz, Waleska Dias Schwarcz, Renata Carvalho Pereira, Adriana de Souza Azevedo, Ana Maria Bispo de Filippis, Clara Lucy de Vasconcellos Ferroco, Geovanna de Lima Cunha Pizzini, Ricardo Cristiano de Souza Brum, Leonardo Secundino, Maria de Fátima de Sousa Andrade, Raquel de Vasconcellos Carvalhaes de Oliveira, Marisol Simões, José Cerbino-Neto, Olindo Assis Martins-Filho, Ana Carolina Campi-Azevedo, Sheila Maria Barbosa de Lima, Luiz Antonio Bastos Camacho, Collaborative Group for Yellow Fever Vaccine Studies

**Affiliations:** 1 Departamento de Epidemiologia, Escola Nacional de Saúde Pública Sergio Arouca (ENSP), Fiocruz, Rio de Janeiro-RJ, Brasil; 2 Departamento de Assuntos Médicos, Estudos Clínicos e Vigilância Pós-Registro (DEAME), Bio-Manguinhos: Instituto de Tecnologia em Imunobiológicos, Fiocruz, Rio de Janeiro-RJ, Brasil; 3 Departamento de Obstetrícia e Ginecologia, Centro de Ciências Médicas (CCM), Universidade Federal da Paraíba, João Pessoa, Paraíba, Brasil; 4 Laboratório de Análise Imunomolecular (LANIM), Bio-Manguinhos: Instituto de Tecnologia em Imunobiológicos, Fiocruz, Rio de Janeiro-RJ, Brasil; 5 Laboratório de Arbovírus e Vírus Hemorrágicos (LARBOH), Instituto Oswaldo Cruz (IOC), Fiocruz, Rio de Janeiro-RJ, Brasil; 6 Laboratório de Epidemiologia Clínica. Instituto Nacional de Infectologia Evandro Chagas (INI), Fiocruz, Rio de Janeiro-RJ, Brasil; 7 Assessoria de Inteligência Competitiva (AICOM), Bio-Manguinhos, Fiocruz, Rio de Janeiro-RJ, Brasil; 8 Laboratório de Pesquisa em Imunização e Vigilância em Saúde (LIVS), Instituto Nacional de Infectologia Evandro Chagas (INI), Fiocruz, Rio de Janeiro-RJ, Brasil; 9 Laboratório de Biomarcadores de Diagnóstico e Monitoração (LDBM), Instituto René Rachou, Fiocruz, Belo Horizonte-MG, Brasil; 10 Departamento de Desenvolvimento Experimental e Pré-Clínico (DEDEP), Bio-Manguinhos: Instituto de Tecnologia em Imunobiológicos, Fiocruz, Rio de Janeiro-RJ, Brasil; Solena Ag, UNITED STATES OF AMERICA

## Abstract

In 2013, the World Health Organization (WHO) withdrew the recommendation of booster doses of the yellow fever (YF) vaccine, based on retrospective and cross-sectional studies that showed lifelong protective immunity from a single dose. Currently, yellow fever transmission in Brazil occurs only through the jungle (sylvatic) cycle. However, the high vector density of *Aedes aegypti*, which transmits other orthoflaviviruses, is a concern for the expansion of YF in other regions of the country. We conducted a cohort study to assess the duration of vaccine-induced immunity in adults and children residing in an area without wild-type YF virus circulation but with a high incidence of other orthoflaviviruses. This phase IV, uncontrolled cohort study was conducted in three municipalities in northeastern Brazil. The 17DD strain vaccine was administered to children aged 9 months to 4 years and adults aged 18 to 50 years. Blood samples for antibody titration were collected before vaccination, 30–45 days after, and one year after vaccination. The following assays were used: µFRNT for yellow fever and dengue; PRNT for Zika; and chemiluminescence for Zika (IgG and IgM) and dengue (IgG). YF seroconversion rates 30–45 days post-vaccination increased with age, reaching 99% in adults, while 10% of infants remained without detectable antibodies. Seropositivity for dengue neutralizing antibodies was inversely associated with YF antibody titers 30–45 days post-vaccination. Previous Zika infection showed no substantial association with YF antibody titers post-vaccination. One year after vaccination, a considerable reduction in YF antibody titers was observed across all age groups, regardless of prior dengue or Zika infections. Our data support the Brazilian National Immunization Program’s recommendation for a booster dose of the vaccine at 4 years of age. Current recommendations assuming lifelong protection from a single dose of the YF vaccine do not appear to provide sufficient protection in high-risk areas, particularly where infants are the primary target for vaccination.

## Author summary

Yellow fever (YF) is a viral disease that can be severe and has caused thousands of deaths in Sub-Saharan Africa, South and Central America. Humans are infected by *Haemagogus* mosquitoes in forests and savannahs, and in urban areas by the *Aedes aegypti*, which also transmits dengue and Zika. These viruses belong to the same genus of the YF virus, and their similarities may confound antibody test results. Protection by vaccines is correlated to antibody levels, which inform about the duration of immunity. Cases of YF in vaccinated individuals are uncommon and result from waning of immunity. Bites by infected mosquitoes boost post-vaccination immunity, while averting disease. Follow-up of vaccinated individuals in a population free from interference of natural YF infection has shown that 30–45 days after vaccination adults had high YF antibody levels, which decreased substantially after one year. In children, post-vaccination antibody levels were lower than in adults and decreased substantially after one year. Waning of immunity was not affected by previous infections with dengue or Zika. These results support the recommendation of a booster dose in populations at risk of YF, especially in children. Routine mass testing after vaccination is not feasible, whereas revaccination is cheap, safe and effective.

## Introduction

Yellow fever (YF) is an infectious disease, caused by the arbovirus yellow fever virus-17D (X03700), species *Orthoflavivirus flavi*, genus *Orthoflavivirus* [1], which has two epidemiological cycles: sylvatic (jungle) and urban. In its sylvatic cycle, non-human primates are the main virus reservoir and transmission occurs through wild vectors, with humans being an accidental host. In the urban cycle, humans are the only host with epidemiological importance and transmission occurs through infected urban vectors, primarily *Aedes aegypti* [2].

In Brazil, the main vectors involved in the sylvatic cycle are *Haemagogus janthinomys* and *Haemagogus leucocelaenus.* Despite the ubiquitous spread of *Aedes aegypti* the urban cycle of the disease has not been recorded in Brazil since 1942 [3]. Sporadic cases and small outbreaks were reported in the Amazonian states and gradually expanded to Southeast (Minas Gerais and São Paulo, respectively in 2002 and 2008) and South (Paraná and Rio Grande do Sul in 2008) [3]. However, between 2016 and 2018 there was a large epidemic that also involved the sylvatic cycle in urban areas of the southeastern region of the country. During this period, 2,276 epizootics were recorded, and 2,153 YF human cases and more than 500 human deaths were confirmed [4]. In Brazil, non-human primates are less resistant to YF infection than in Africa, so epizootic surveillance is a good marker of virus circulation. The most affected monkeys are of the genus *Alouatta*, *Sapajus* and *Callithrix*. The sizable number of *Callithrix* epizootics related to the latest outbreak in Brazil was concerning because this species of primates is abundant in urban areas [5]. This epidemic did not characterize the reurbanization of YF, due to the presence of epizootics, but the increase in human cases in areas with high vector density of *Aedes aegypti* was decisive for the expansion of vaccination in the affected states [6].

The YF vaccine is the most important measure for the prevention and control of the disease in man. Currently available vaccines are highly immunogenic, inducing serum protection of approximately 98% in adults [7]. Children present lower seroconversion rates than adults, especially those aged between 9 and 23 months, whose seroconversion 30 days after vaccination can be as low as 85.8% [8]. Antibody levels are determinant for the duration of immunity, as higher antibody titers persisted for more than 10 years while lower titers decreased after 3 years [9].

In 2013, the World Health Organization (WHO) withdrew the recommendation of booster doses of the YF vaccine based on a systematic review conducted by Gotuzzo *et al.* [10], which indicated that the duration of vaccine-induced immunity could be sustained throughout life [11]. That was not consistent with a study in Brazil in which the geometric mean titers for YF 30–45 days after vaccination declined to approximately 81% 10 years after vaccination in adults with 1 dose of the vaccine [12]. Moreover, in children who had been vaccinated at 9 months of age the proportion seropositive 2–5 years after vaccination varied from 27.8% to 50.4% [13].

Currently, the National Immunization Program of the Brazilian Ministry of Health establishes that the vaccination schedule consists of a dose of the vaccine at 9 months of age and a booster at 4 years. Individuals between 5 and 59 years of age should receive a dose of the vaccine, and those aged 60 years or older should assess the risk of infection with the risk of post-vaccination adverse events [14].

In addition to these uncertainties related to the duration of vaccine immunity, the control of YF has other challenges related to *Aedes aegypti,* the potential transmission vector, as it is the same for other relevant arboviruses in Brazil: dengue and Zika. Considering the high density of *Aedes* mosquitoes in many Brazilian urban areas where vaccination coverage is low the risk resurgence of YF urban transmission is not negligible [15].

YF, dengue and Zika belong to the same family of viruses, *Flaviviridae*, and their antigenic similarity could cause cross-reaction in serological tests and plausibly affect the immune response to YF vaccine. The possible cross-reaction between YF and dengue was found in the association between the presence of dengue antibodies and the severity of YF. However, no association with infection was found [16]. Regarding the impact on the post-YF vaccination immune response, it was demonstrated that cellular immune response was not influenced by seropositivity for dengue and/or Zika [17].

Given this context, this study aims to advance the understanding of the duration of immunity induced by the YF vaccine in adults and children living in an area without circulation of wild-type YF virus, but with a high incidence of other orthoflaviviruses. In endemic areas natural YF infection in vaccinated individuals act as a booster, thus hampering the assessment of the duration of immunity by the vaccine. The study also intends to contribute to the evaluation of possible interferences in the immune response due to dengue and/or Zika infections detected by neutralizing antibodies.

## Materials and methods

### Ethic statements

The study followed the requirements of the Declaration of Helsinki [18] and Resolution 466 of the Brazilian National Health Council [19]. Formal written consent was obtained from all participants. For child participants written formal consent was obtained from the parent/guardian. The study protocol was approved by the Institutional Review Board of Federal University of Paraíba (CAAE: 51160215.7.3001.5183). A Data Monitoring Committee combined the expertise of four senior professionals in vaccines, immunization program and clinical research (an epidemiologist, a coordinator of state immunization program, an advisor of the Brazilian National Immunization Program, and an expert in adverse events following immunization) provided independent assessment of the safety, scientific validity and integrity of the study.

### Design, sampling, area, and study period

This is a phase IV study, in an uncontrolled cohort, conducted in the municipalities of Alhandra (20,000 inhabitants), Caaporã (22,000 inhabitants) and Conde (25,000 inhabitants), state of Paraíba, located in the Northeast region of Brazil. This state was selected because there was no circulation of the YF virus in the region, nor recommendation for routine vaccination of the local population at the time of the beginning of the study. Moreover, outbreaks of dengue and Zika had been reported in that area. The municipalities were chosen for logistical reasons, such as ease of transportation of vaccines and blood samples to the state capital, cooperative personnel to reach out for participants and formal agreement of municipal authorities to allow the 10-year study follow-up. Implementation of field work in those municipalities required a painstaking preparation of equipment and personnel. Sustaining adherence to the study protocol was challenging and demanded intense scrutiny from clinical monitors and frequent visits by study coordinators. Follow-up of participants who missed appointments often required visits to their residences, some of them in rural areas distant from the city center. Community health workers were very helpful in contacting participants in general and finding out the addresses of those who moved out of the municipality. Informing participants of the serological test results was critical to reduce withdrawals. Meetings with health professionals and health authorities were conducted to explain the study objectives and methods and to provide feedback on the field work. YF vaccination and follow-up began in July 2016 and participant enrollment was completed in July 2017. The results presented here are based on pre-vaccination serological tests, and address the main study outcome, namely, serological status for yellow fever, 30–45 days and 1 year post-vaccination.

A sample of 2,756 children was estimated, assuming 50% will remain seropositive at the end of the study. For adults, a sample of 2,005 was estimated, considering the 76% seropositivity observed 10 years after one dose of YF vaccine in a sectional study [12]. Sample sizes considered a 95% confidence level, 3% error for global estimates, and 10% loss to follow-up.

Participants were recruited from individuals who spontaneously attended health units or who were encouraged by community health agents to participate in the study. The primary health units were selected to participate in the study in agreement with the health secretaries of the municipalities of Alhandra, Conde and Caaporã.

### Inclusion and exclusion criteria in the study

The inclusion criteria of the participants in the study were: having a permanent address in one of the 3 municipalities participating in the study; age from 9 months to 4 years and from 18 to 50 years at the beginning of the follow-up; and medical evaluation without significant findings. The rationale for targeting those age subgroups were: YF vaccination in Brazil is recommended for 9-month-old infants, and a booster dose is recommended at 4 years of age. This age subgroup has been shown to have suboptimal immune response compared to adults. Individuals above 60 years of age are considered more susceptible to severe adverse events, which was a major concern in an area where routine YF vaccination was not recommended. We set the upper limit for inclusion at 50 years of age considering that with the 10-year follow-up, a safety margin against interference of immunosenescence was needed.

The exclusion criteria defined for the study were: previous YF vaccination; having resided in or traveled to endemic areas or where YF outbreaks have occurred; having received or having to receive the MMR or MMRV vaccine (measles-mumps-rubella-varicella combination) within 30 days; presumed or confirmed pregnancy, at any stage, at the time of vaccination; breastfeeding; HIV infection; use of immunosuppressive medications; history of anaphylactic reaction to any of the vaccine components; autoimmune diseases; history of thymus diseases; treatment with immunoglobulin, blood or derivatives transfusions up to 60 days prior to vaccination; and acute febrile illness, with impairment of general health.

### Vaccination procedures

Study participants received the YF vaccine produced by Bio-Manguinhos (Fiocruz, Brazil) from the attenuated 17DD strain of the YF virus. This has been the only YF vaccine available in the National Immunization Program of the Brazilian Ministry of Health. Vaccine Lots 172VFA002Z (4.99 log_10_ pfu/dose), 172VFA002Z (4.91 log_10_ pfu/dose) and 174VFA035Z (4.63 log_10_ pfu/dose) were used in this study. The vaccinators were trained by the Bio-Manguinhos Clinical Advisory team in the management and application of the YF vaccine, since this vaccine was not part of the vaccination routine of the health units participating in the study. Administration was subcutaneous, preferably in the deltoid region, the outer face of the upper right arm, or the upper outer quadrant of the gluteal region. The storage and transport of vaccine batches followed National Immunization Program standards.

Monitoring of adverse events following vaccination was established to ensure medical care for immediate events while participants remained under observation in the health unit, as well as hospital care for severe events, regardless of causality, in a University Hospital in the state capital city of João Pessoa (the capital city of the state where field work took place). We recorded all signs and symptoms reported 10 days (via telephone contact) and 45 days after vaccination (in a questionnaire). Causality assessment of severe adverse events (leading to hospitalization, death or sequelae) following immunization was based on medical records and patient information analyzed by study coordinators and reported to the Data Monitoring Committee.

### Laboratory methods

YF neutralizing antibodies were titrated by micro focus-reduction neutralization test (YFV μFRNT), carried out at Virological Technology Laboratory, and Immunomolecular Analysis Laboratory (LATEV and LANIM, respectively, Bio-Manguinhos, Fiocruz-Rio de Janeiro, Brazil), according to Simões et al. [20]. Briefly, in 96-well plates, a mixture of serial diluted serum samples (1:6–1:1,458) plus approximately 70 PFU/well of the 17D-213/77 vaccine virus was incubated for 2 hours at 37 °C, 5% CO_2_. After incubation, mixtures were transferred to another 96-well plate containing preformed Vero ATCC cell monolayer for adsorption during 1 hour at 37 °C, 5% CO_2_. The mixtures were removed, plates were washed and replaced by 2% Carboxymethylcellulose semisolid medium (CMC) followed by incubation for 48 hours at 37 °C, 5% CO_2_. Thereafter, the monolayers were washed and fixed with ethanol/methanol (1:1) solution for 1 hour and 30 minutes at −20 °C. After fixation, the HRP-conjugated monoclonal antibody (4G2) diluted in the blocking solution (5% of BSA 10%; 50% of Blocker Casein in PBS 1%; 45% of distilled water) was added to each well and plates incubated for 2 hours at 35 °C and 5% CO_2_. Subsequently, cell monolayers were washed with Dulbecco’s Phosphate-Buffered Saline (DPBS) before addition of True Blue Dye (TB) substrate. After 15 minutes of incubation in the dark at room temperature, the cell monolayers were washed, dried and photographed using the automated image acquisition system according to Denani et. al. [21]. The antibody titers were determined considering the endpoint as the last serum dilution that reduced the number of lysis plaques by 50% (EP_50_) as compared to control.

Antibody titers (reciprocal dilution) were categorized as: seronegative (titers ≤70); indeterminate (titers ≥71 and <100; and seropositive (titers ≥100). Subjects who did not seroconvert, that is, remained seronegative or were indeterminate 30–45 days after vaccination, were offered a 2nd dose of the YF vaccine and a new serological test was performed. In these cases, if there was still no seroconversion, another dose of the vaccine was not applied, and the participant was considered a non-responder. These participants were kept in the study, following the collection schedules of the other participants, with reference to the date of the 1st vaccination.

Serological analysis for dengue were performed with IgG Panbio [22] and Euroimmun [23]. These test kits have antigen-coated wells that bind to the specific antibodies in positive samples. These serological analyses were performed at the Immune Technology Laboratory (LATIM, Bio-Manguinhos, Fiocruz-Rio de Janeiro, Brazil). Serology for Zika was performed by immunochromatographic tests (IgM and IgG) manufactured by Bio-Manguinhos, in which the test support has 2 windows that identify the reaction between the antigen and the IgM and IgG antibodies separately [24]. These serological analyses were performed at the Arbovirus and Hemorrhagic Virus Laboratory at Instituto Oswaldo Cruz (LARBOH, Fiocruz-Rio de Janeiro, Brazil).

The titration of neutralizing antibodies for dengue (μFRNT) and Zika (PRNT_90_) was also performed in LATEV/LANIM in non-probabilistic subsamples of 764 and 711 participants in the pre-vaccination and 30–45 days post-vaccination time points, respectively. For our analyses we considered neutralizing antibodies for any of the 4 serotypes of the dengue virus.

The Zika PRNT_90_ result considered the categories: seronegative (titers ≤1:139) and seropositive (titers ≥1:140). While the dengue µFRNT considered as cutoff points: seronegative (titers ≤1:29); and seropositive (titers ≥1:30).

### Exploratory analysis

A descriptive analysis of the study population considered the variables sex, skin color/race, education, income, occurrence of adverse events, and seropositivity for YF, dengue and Zika. These were the variables of interest in a study group comprising individuals free of major health problems generally considered contraindications for YF vaccine. These analyses will be presented in tables with absolute and relative frequencies and, when necessary, separated by age group (children, between 9 months and 4 years, and adults, 18–50 years) or age range (9–23 months, 2–4 years, 18–35 years, 36–50 years).

In addition, the percentage of yellow fever seroconversion and the geometric means of the antibody titers for YF and their respective 95% confidence intervals were evaluated, according to the serological status of dengue and Zika. This way, the assessment of the qualitative and quantitative immune responses took into consideration previous infections with the two other orthoflavivirus of public health concern in Brazil. The Person’s chi-square test was applied to test the association between the serological status for YF, Zika, and dengue at three different time points (pre-vaccination, 30–45 days, and 1-year post-vaccination).

For the analysis of seroconversion the antibody titers for YF classified as indeterminate, according to the established cutoff point, were grouped with those classified as seronegative, thus excluding only the seropositive ones. However, for the analyses with IgG serologies for dengue and Zika, individuals with indeterminate results were excluded due to the possibility of classification error. The handling of the *indeterminate* serological status had a conservative approach to safeguard the analyses against the uncertainty of that category.

### Statistical modelling

Categories of low and high post-vaccination YF antibody titers were defined, for children and adults, as cutoff values based on significant changes in the distribution of YF antibody titers, by segmented (joinpoint analysis from the package segmented from the R software [25]) of generalized linear model (glm) with the Gamma distribution. The glm model considered the YF antibody titer as the outcome, with non-normality distribution, and the key participant index as a covariate. The joinpoint analysis estimates the point of the change in the effects (slopes) on the YF antibody titers. In order to evaluate seroconversion for YF, individuals seropositive for YF before vaccination and individuals seronegative or with indeterminate titers 30–45 days after vaccination were excluded. In the analysis with the segmented model of the sample of participants who had IgG serology for dengue and Zika, the reciprocal dilution of YF antibody titers among children was classified as low (≤ 856) and high (>856). For adults, the cutoff point was set at 951. Analysis with the segmented model was also conducted in the subsample of individuals who had their sera tested for neutralizing antibodies for dengue (μFRNT) and Zika (PRNT_90_): the cutoff point of YF antibody levels (reciprocal dilution) was 912 for children, and 1,106 for adults (S1 Fig).

We performed a binary logistic regression model (with logit link function) of the intensity (low and high) of the immune response 30–45 days after vaccination. The effects of pre-vaccine serological status for dengue and Zika were obtained in models adjusted for sex and age (in years for adults and months for children). The crude and adjusted Odds Ratios (OR) were provided, with the respective confidence intervals (95% CI), for the single models and the multicovariate models, respectively. Akaike Information Criterion (AIC) verified the goodness of the models’ fit. Statistical analyses were performed in the R software (version 4.2.2) [26].

## Results

**Fig 1:**
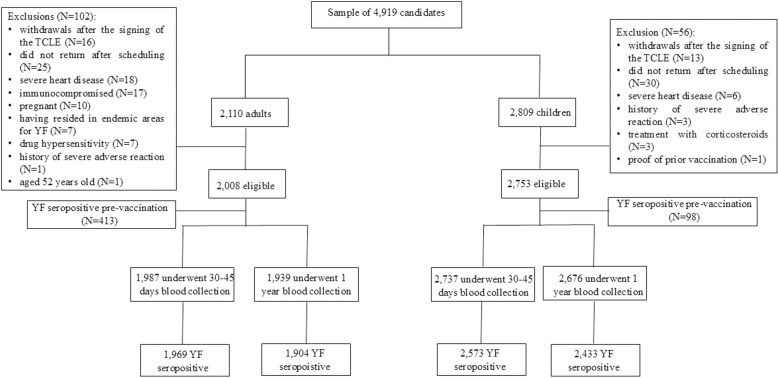
Diagram of study participants and blood samples pre-vaccine, 30–45 days and 1 year after vaccination. The collections are not exclusive, that is, individuals who missed a follow-up contact were still eligible for subsequent blood collections.

Only 3.2% of the candidates did not meet the eligibility criteria for the study. Blood collection 30–45 days after vaccination was performed in 99.0% of adults and 99.4% of children. Blood collection 1 year after vaccination was performed in 96.6% of adults and 97.2% of children (Fig 1).

Approximately 77% of the adults and 50% of the participating children were female (Table 1). Children younger than 2 years represented 30.7%, and in this subgroup 26.2% were younger than 1 year, with a median age of 10 months. Subjects between 18 and 35 years of age represented 63.8% of adults. The predominant self-reported skin color/race was brown, and most adults, whether participants themselves or guardians of participating children, had no source of income. Almost 40% of adults (including children’s parents or guardians) had not completed elementary schooling.

**Table 1:** Sociodemographic and pre-vaccination status for yellow fever, dengue and Zika by age group.

Variables	Age group			
	Children		Adults	
	n	%	n	%
**Sample**	2,753	57.8	2,008	42.2
**Sex**				
Female	1,365	49.6	1,543	76.8
Male	1,388	50.4	465	23.2
**Skin color/race**				
White	443	16.1	251	12.5
Yellow	9	0.3	22	1.1
Brown	2,177	79.1	1,495	74.5
Black	104	3.8	162	8.1
Indigenous	0	0.0	3	0.1
Other	20	0.7	75	3.7
**Education** ^*^				
Illiterate	59	2.1	44	2.2
Incomplete elementary school	1,107	40.2	750	37.3
Complete elementary school	606	22.0	384	19.1
Complete high School	904	32.9	740	36.9
Complete college/graduate	77	2.8	90	4.5
**Source of income** ^*^				
Retired	6	0.2	12	0.6
Employed	341	12.4	551	27.4
Self-employed	199	7.2	162	8.1
Student	22	0.8	78	3.9
Other	87	3.2	176	8.8
No source of income	2,098	76.2	1,029	51.2
**Municipality of residence**				
Alhandra	238	8.6	570	28.4
Caaporã	1,250	45.4	572	28.5
Conde	1,265	46.0	866	43.1
**Serological status for YF (µFRNT)**				
Negative	2,534	92.0	1,423	70.8
Indeterminate	120	4.4	171	8.5
Positive	98	3.6	413	20.6
Unavailable^**^	1		1	
**Serological status for dengue (IgG)**				
Negative	2,299	83.5	209	10.4
Indeterminate	151	5.5	35	1.7
Positive	279	10.1	1,762	87.7
Unavailable^**^	24	0.9	3	0.2
**Serological status for Zika (IgG)**				
Negative	2,376	86.3	635	31.6
Indeterminate	6	0.2	31	1.55
Positive	367	13.3	1,342	66.8
Unavailable^**^	4	0.2	1	0.05
**Serological status for Zika (IgM)**				
Negative	2,736	99.4	1,985	98.8
Indeterminate	1	0.04	0	0.0
Positive	12	0.4	23	1.1
Unavailable^**^	4	0.16	1	0.1

* For children, information refers to mothers or guardians.

** Some results were unavailable due to insufficient sample volume for laboratory analysis.

A substantial and unexpected proportion of participants, especially adults, were seropositive for YF before vaccination. More than 87% of adults and 10% of children were seropositive for dengue (IgG) pre-vaccination. These seropositivity rates were similar for Zika (IgG), but recent infections of Zika (IgM) were scarce (Table 1).

Ascertainment of adverse events was done through telephone contacts between 9 and 12 days after vaccination in 98.5% of the research participants. 3.8% and 2.8% of adults and children, respectively, had some sign or symptom, temporally associated with vaccination. Eight participants had severe adverse events, whose causality was assessed: two deaths from trauma and one death from myocardial infarction (eight months after vaccination), two hospitalizations for trauma with successful treatment, one hospitalization for neuroblastoma (4 months after vaccination) and two hospitalizations for treatment of viral meningitis. These two cases of meningitis were temporally related to vaccination (21 and 17 days after vaccination) and were successfully managed. Both were discharged from hospital with no sequelae.

### Immune response to yellow fever vaccination

The seropositivity rates after 30–45 days of vaccination increased with age in all municipalities, reaching almost all adults but leaving a considerable proportion of children with no detectable response to vaccination (S1 Table). After 1 year the proportions of seropositivity decreased slightly in children and even less in adults.

**Fig 2:**
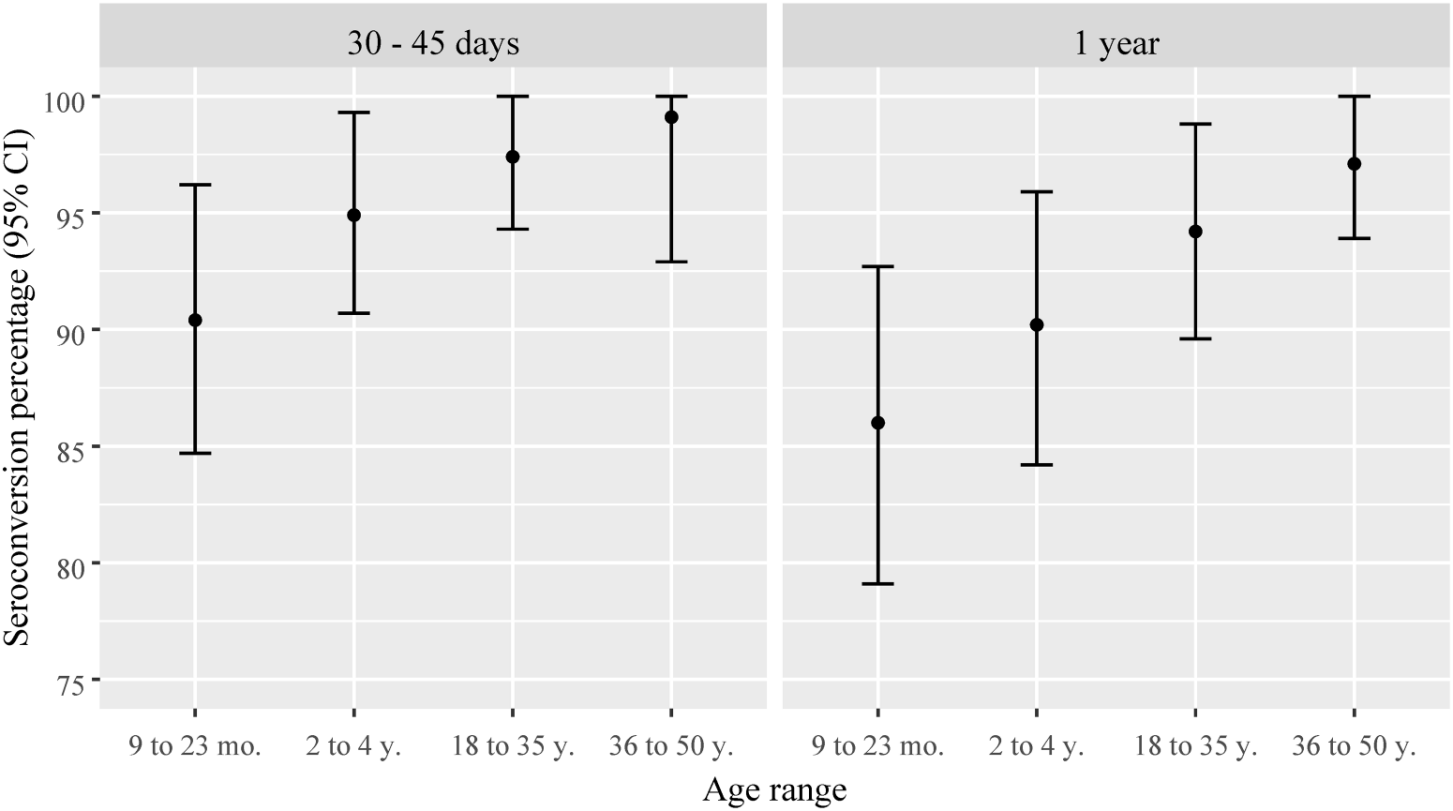
Percentage and 95% confidence intervals of yellow fever seroconversion 30–45 days and 1 year after vaccination by age range. Pre-vaccine yellow fever seropositive individuals excluded.

**Fig 3:**
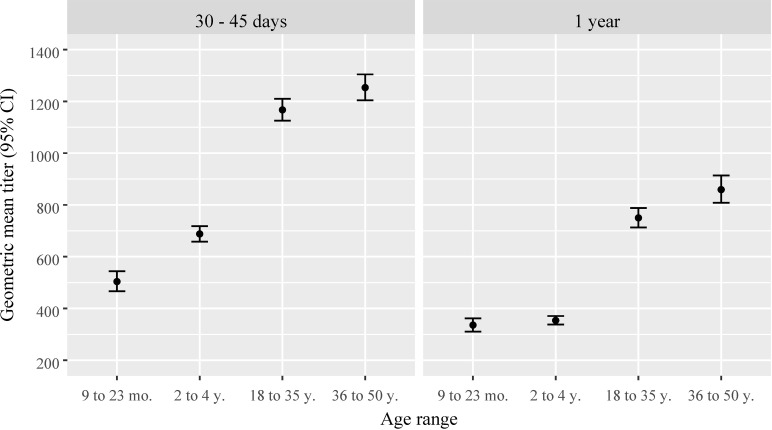
Geometric means and 95% confidence intervals of yellow fever antibody titers (reciprocal of dilution) 30–45 days and 1 year after vaccination by age range. Pre-vaccine yellow fever seropositive individuals excluded.

Disregarding those participants seropositive before vaccination, the proportions of YF seroconversion after 30–45 days of vaccination increased with age, reaching 99% of adults in the three municipalities; only 1 adult remained seronegative. However, among the children, 15 remained seronegative and 8 presented undetermined YF μFRNT results. One year after vaccination, a slight reduction in seroconversion proportions was observed, especially in children (Fig 2). The geometric mean of YF antibody titers followed a similar pattern, with higher titers observed in adults. However, a substantial reduction in antibody titers was observed across all age ranges 1 year after vaccination. The 95% confidence intervals indicated statistical significance when compared to titers measured 30–45 days after vaccination (Fig 3).

### Immune response to yellow fever vaccination and association with serological status for dengue and Zika

YF seropositivity 30–45 days after vaccination was slightly higher in participants who were seropositive for dengue and Zika, both before and after YF vaccination (S2 Table). Higher YF seropositivity was also observed 1 year after vaccination in participants seropositive for dengue and Zika, both 30–45 days and 1 year after YF vaccination (S3 Table). Although differences in YF seropositivity across dengue and Zika subgroups were small, they were statistically significant.

In the subsample with serological status for dengue and Zika determined by neutralizing antibodies (dengue μFRNT and Zika PRNT90), the proportion of seroconversion in both children and adults 30–45 days after vaccination did not differ significantly between seronegative and seropositive individuals for dengue, Zika, or both prior to vaccination (Tables 2 and 3).

**Table 2:** Distribution of children (between 9 months and 4 years) by serological status for yellow fever 30–45 days after vaccination, stratified by pre-vaccine serological status (neutralizing antibodies) for dengue and Zika.

Variables	μFRNT yellow fever 30–45 days						p-value
	Seronegative		Indeterminate		Seropositive		
	n	%	n	%	n	%	
**μFRNT dengue pre-vaccination**							0.609
Negative	6	3.2	3	1.6	178	95.2	
Positive	9	5.1	5	2.9	161	92.0	
**PRNT**_**90**_ **Zika pre-vaccination**							0.875
Negative	9	4.6	5	2.6	181	92.8	
Positive	6	3.6	3	1.8	158	94.6	
**μFRNT dengue and PRNT**_**90**_ **Zika pre-vaccination**							0.976
At least 1 negative	3	4.2	2	2.8	67	93.0	
Negative	6	3.9	3	1.9	146	94.2	
Positive	6	4.4	3	2.3	126	93.3	

Pre-vaccine yellow fever seropositive individuals excluded.

Data from the subsample of participants tested for μFRNT dengue and PRNT_90_ Zika.

**Table 3:** Distribution of adults (between 18 and 50 years) by serological status for Yellow Fever 30–45 days after vaccination, stratified by pre-vaccine serological status (neutralizing antibodies) for dengue and Zika.

Variables	μFRNT yellow fever 30–45 days					
	Seronegative		Indeterminate^*^		Seropositive	
	n	%	n	%	n	%
**μFRNT dengue pre-vaccination**						
Negative	0	0.0	–	–	82	100
Positive	1	0.6	–	–	158	99.4
**PRNT**_**90**_ **Zika pre-vaccination**						
Negative	1	0.5	–	–	218	99.5
Positive	0	0.0	–	–	22	100
**μFRNT dengue and PRNT**_**90**_ **Zika pre-vaccination**						
At least 1 negative	1	0.7	–	–	140	99.3
Negative	0	0.0	–	–	80	100
Positive	0	0.0	–	–	20	100

Pre-vaccine yellow fever seropositive individuals excluded.

Data from the subsample of participants tested for μFRNT dengue and PRNT_90_ Zika.

* Unable to calculate p-values due to low count of 2 categories of the variable.

### Geometric mean of YF antibody titers stratified by dengue and Zika serological status (neutralizing antibodies) at the different timepoints

In the subsample of children with available neutralizing antibody titers for Zika and dengue (S4 Table), YF-GMT 30–45 days after vaccination was higher in participants who were seronegative (µFRNT) for dengue prior to vaccination. YF-GMT did not appear to be influenced by pre-vaccine seropositivity for Zika, nor by combined seropositivity for dengue and Zika. No apparent association was found between YF-GMT and combined dengue and Zika seropositivity 30–45 days after YF vaccination. One year after vaccination, children who were seronegative for dengue at the 30–45 day timepoint had a higher YF-GMT. Similarly, children seronegative for both dengue and Zika at the 30–45 day timepoint also had higher YF-GMT.

In the subsample of adults with neutralizing antibody titers for Zika and dengue, YF-GMT 30–45 days after vaccination was higher in the subgroup that was seronegative for dengue before vaccination (S5 Table). This pattern persisted for YF-GMT 1 year after vaccination, with neutralizing antibodies for both dengue and Zika 30–45 days after YF vaccination. The decrease in YF GMT one year after vaccination was more pronounced in children and adults who were seropositive for both dengue and Zika.

### Association of pre-vaccination dengue and Zika serological status (IgG antibodies) and intensity of immune response 30–45 days after yellow fever vaccination

For YF seronegative children, who seroconverted 30–45 days after vaccination, the adjusted regression coefficient (0.74 with “marginal” statistical significance) suggested that dengue IgG seropositive children were 26% less likely to have higher levels of YF neutralizing antibodies (Table 4). Likewise, the odds of Zika-seropositive (IgG) children before YF vaccine administration having high antibody titers of YF 30–45 days after vaccination was 32% lower than Zika-seronegative children, adjusted for sex, age (in months), and pre-vaccine dengue IgG. The odds of presenting high antibody titers of YF increased 2 percentage points with each 1-month increase in age, adjusted for sex, dengue (IgG) and Zika (IgG) before YF vaccination.

**Table 4:** Logistic models to high^*^ neutralizing antibody titer for yellow fever 30–45 days after vaccination in children (between 9 months and 4 years) (n = 2,328).

Variables	Crude OR (95% CI)	Adjusted OR^**^ (95% CI)
**IgG dengue pre-vaccination**		
Positive	0.76 (0.58–0.99)	0.74 (0.54–1.02)
Negative	1	1
**IgG Zika pre-vaccination**		
Positive	0.80 (0.62–1.03)	**0.68 (0.50–0.93)**
Negative	1	1
**Age (months)**	**1.02 (1.01–1.02)**	**1.02 (1.01–1.03)**

Pre-vaccine yellow fever seropositive individuals and individuals who had seronegative and indeterminate titers 30–45 days post-vaccination were excluded.

* Reference category: μFRNT YF ≤ 856 (low).

** Model adjusted by IgG dengue pre-vaccination, IgG Zika pre-vaccination, Age (months) and sex: AIC = 3,145.

For adults that seroconverted for YF 30–45 days after vaccination, dengue seropositives (IgG) before YF vaccination were 59% less likely to have higher levels of YF neutralizing antibodies, adjusted for sex, age and pre-vaccine Zika IgG (Table 5). The odds of presenting high antibody titers of YF increased 2 percentage points with each 1-year increase in age, adjusted for sex, dengue (IgG) and Zika (IgG) before YF vaccination.

**Table 5:** Logistic models to high^*^ neutralizing antibody titer for yellow fever 30–45 days after vaccination in adults (between 18 and 50 years) (n = 1,504).

Variables	Crude OR (95% CI)	Adjusted OR^**^ (95% CI)
**IgG dengue pre-vaccination**		
Positive	**0.45 (0.23–0.79)**	**0.41 (0.21–0.75)**
Negative	1	1
**IgG Zika pre-vaccination**		
Positive	0.92 (0.66–1.28)	1.12 (0.79–1.56)
Negative	1	1
**Age (year)**	**1.02 (1.00–1.04)**	**1.02 (1.00–1.04)**

Pre-vaccine yellow fever seropositive individuals and individuals who had seronegative and indeterminate titers 30–45 days post-vaccination were excluded.

* Reference category: μFRNT YF ≤951 (low).

** Model adjusted by IgG dengue pre-vaccination, IgG Zika pre-vaccination, Age (years) and sex: AIC = 1,108.

### Pre-vaccination dengue and Zika neutralizing antibodies and intensity of immune response 30–45 days after yellow fever vaccination

In the subsample of participants who underwent pre-vaccine dengue μFRNT and Zika PRNT_90_, both in children (Table 6) and adults (Table 7), dengue neutralizing antibodies were inversely associated with levels of antibody YF titers 30–45 days after vaccination, with statistical significance. Conversely, Zika neutralizing antibodies did not show statistically significant association with YF antibody levels. The odds of dengue-seropositive (neutralizing antibodies) adults before YF vaccine administration having high antibody titers of YF 30–45 days after vaccination was 66% lower than a dengue-seronegative adult adjusted for sex, age, and pre-vaccine Zika PRNT_90_. In children, the odds of presenting high antibody titers of YF were 43% lower. In adults, age did not show statistically significant, but in children the odds of presenting high antibody titers of YF increased 2 percentage points with each 1-month increase in age, adjusted for sex, dengue μFRNT and Zika PRNT_90_.

**Table 6:** Logistic models to high^*^ neutralizing antibody titer for yellow fever 30–45 days after vaccination in children (between 9 months and 4 years) (n = 339).

Variables	Crude OR (95% CI)	Adjusted OR^**^ (95% CI)
**μFRNT dengue pre-vaccination**		
Positive	0.72 (0.47–1.11)	**0.57 (0.32–0.99)**
Negative	1	1
**PRNT**_**90**_ **Zika pre-vaccination**		
Positive	0.95 (0.61–1.45)	1.04 (0.59–1.85)
Negative	1	1
**Age (months)**	1.01 (0.99–1.03)	**1.02 (1.00–1.04)**

Pre-vaccine yellow fever seropositive individuals and individuals who had seronegative and indeterminate titers 30–45 days post-vaccination were excluded; Data from the subsample of participants tested for μFRNT dengue and PRNT_90_ Zika.

* Reference category: μFRNT YF ≤ 912 (low).

** Model adjusted by μFRNT dengue pre-vaccination, PRNT_90_ Zika pre-vaccination, Age (months) and sex: AIC = 468.

**Table 7:** Logistic models to high^*^ neutralizing antibody titer for yellow fever 30–45 days after vaccination in adults (between 18 and 50 years) (n = 240).

Variables	Crude OR (95% CI)	Adjusted OR^**^ (95% CI)
**μFRNT dengue pre-vaccination**		
Positive	**0.35 (0.15–0.76)**	**0.34 (0.14–0.74)**
Negative	1	1
**PRNT**_**90**_ **Zika pre-vaccination**		
Positive	1.51 (0.49–6.64)	2.06 (0.64–9.22)
Negative	1	1
**Age (year)**	1.02 (0.98–1.07)	1.02 (0.98–1.06)

Pre-vaccine yellow fever seropositive individuals and individuals who had seronegative and indeterminate titers 30–45 days post-vaccination were excluded; Data from the subsample of participants tested for μFRNT dengue and PRNT_90_ Zika.

* Reference category: μFRNT YF ≤1.106 (low).

** Model adjusted by μFRNT dengue pre-vaccination, PRNT_90_ Zika pre-vaccination, Age (years) and sex: AIC = 230.

## Discussion

Our results demonstrated a high proportion of seroconversion after YF vaccination, although lower in children aged 9 months to 4 years compared to adults aged 18–50 years. Additionally, antibody levels increased with age, particularly in children aged 2–4 years compared to those aged 9–23 months. However, YF antibody titers decreased 1 year after vaccination in all age groups, regardless of prior infection with other widely circulated orthoflaviviruses. These findings are the first to examine a cohort of vaccinees with serological follow-up in an area without YF viral circulation.

The study was conducted in northeastern Brazil, an area that has remained free of YF in both humans and nonhuman primates, which made it an ideal location for the study. Therefore, interference from natural infection, which could not be ruled out in previous studies, was unlikely in this cohort. Moreover, the few participants who traveled outside the study area would have had a negligible effect on the results, given the very small number of human cases and epizootics in the country since 2018.

These findings align with a previous cross-sectional study, which found that 76.4% of children were seropositive between 9 and 23 months after vaccination [27]. In addition to showing lower seroconversion rates, children aged 9–12 months had significantly lower antibody titers over time, with seropositivity dropping below 60% four years after vaccination [28]. The extent of seroreversion in these studies could not be assessed, as pre- and short-term post-vaccination serological data were unavailable, and seroconversion could not be verified.

Our findings also align with a study of 171 adults aged 18–74 years, which found an inverse correlation between YF neutralizing antibody levels and the time since primary vaccination [29]. In another cross-sectional study of adults who received two doses of the YF vaccine, geometric mean titers between 1 and 5 years after the second dose were approximately 60% of those seen in individuals who had received the vaccine less than a year before [30].

Notably, these cross-sectional studies evaluated different cohorts with varying times since vaccination. Another Brazilian study found a decrease in seropositivity and YF neutralizing antibody levels 10 years after vaccination. However, seropositivity was higher in those who had received a booster dose, suggesting that booster doses are essential for maintaining sufficient antibody levels for protection, particularly in areas with a high risk of transmission [31].

Statistical modeling was used to analyze the independent effects of serological status for dengue and Zika on the immune response intensity to YF vaccination. In adults, previous dengue infection (in all participants with IgG and the subsample with neutralizing antibodies), adjusted for age, sex, and Zika serological status, appeared to decrease the odds of a more intense immune response 30–45 days after YF vaccination. Previous Zika infection showed no significant association in adults. However, children previously infected with Zika (indicated by IgG) appeared less likely to have stronger immune responses, although this was not observed in the subsample with Zika neutralizing antibodies. . These findings were consistent with the greatest reduction in YF antibody levels observed 1 year after vaccination among dengue and Zika seropositive individuals. Altogether, these results suggest that previous infections with both orthoflaviviruses affected the immune response to YF vaccination and possibly the degree of immunity waning after one year.

The potential interference of dengue and Zika serological status in YF antibody titration was negated by the lack of a substantial effect of dengue and Zika serological status before and 30–45 days after YF vaccination. Previous Brazilian cross-sectional studies in the southeastern region found no significant differences in YF post-vaccine seropositivity (PRNT) between dengue seropositive and seronegative (IgG) individuals [12,30]. However, a dose-response study of the 17DD vaccine conducted in Brazil found that post-vaccination YF viremia was more frequent in dengue seronegative individuals [32].

There appears to be a complex interplay between the response to a live attenuated vaccine and antibodies from previous natural infections by antigenically related orthoflaviruses. Antibodies from previous dengue infections could not only limit the intensity of the immune response to YF vaccination but also plausibly affect the accuracy of antibody titration methods. Further analyses are limited by the qualitative results of dengue and Zika (“positive”/ “negative”, rather than antibody titers), and by the ‘ceiling effect’ (cluster of the highest available YF antibody titer), which led to the simplification of titers into ‘high’ and ‘low’ categories. Regardless of the mechanisms of a true association, this empirical evidence of immunological interference may support the justification for booster doses, particularly given the widespread nature of dengue infection in Brazil. The growing availability of dengue vaccines may complicate the management of vaccination calendars in the near future.

At the time of this writing, the authors are unaware of other cohort studies in areas not endemic for YF and endemic for dengue and Zika. The dengue virus has been circulating in Brazil since the 1980’s but Zika was introduced in 2015, and thus, its implications are less well known. Thus, the heightened relevance of this study lies in the administering the vaccine and monitoring the serological status of adults and children for both YF and other orthoflaviviruses of public health importance. Although the serological correlates of protection for YF in humans are not known, the substantial and early reduction in antibody levels after vaccination may indicate the potential for secondary vaccine failure (loss of protection after seroconversion from a single dose), especially in children. The YF vaccine was included in the immunization routine in the whole country in 2020, and study participants were urged not to seek revaccination and had their vaccination cards tagged with the study logo.

On the issue of generalizability of the findings we argue that the duration of immunity genuinely provided by the vaccine, that is, without the interference of natural booster, can inform of the optimal timing for revaccination in endemic areas. As routine serological testing of vaccinees is not feasible, and revaccination is safe and affordable, data on the duration of immunity provided by one dose of the vaccine provide key elements to recommend the need and the timing for a regular booster dose. It may also indicate the need for revaccination in residents or visitors to areas with an ongoing YF outbreak. In fact, the CDC Advisory Committee on Immunization Practices [33], Yellow fever vaccine: Canadian Immunization Guide [34] and UK’s NHS recommend revaccination of those at high risk of exposure (e.g., travelling to an area experiencing an epidemic or major outbreak, or travelling frequently or for prolonged periods to countries with risk of YF transmission.

We acknowledge limitations in the study. The proportion seropositive for YF before vaccination was unexpected and indicated a violation of inclusion criteria. Undeclared previous vaccination probably accounted for most cases, whereas false positives may explain low titers, as those observed in infants. Natural infection seemed very unlikely in those individuals. In any case, data analyses proceeded without those individuals who had YF antibodies before vaccination. Another limitation was the high number of participants with YF antibody titers at the highest test dilution (“ceiling effect”), which limited estimation and comparisons of geometric means, especially in adults, who showed more intense responses. We addressed this limitation by categorizing antibody titers into high and low, for adults and children separately, but differences among subgroups may have been missed.

The logistic models allowed us to verify the effect of dengue infection on the intensity of the response to the YF vaccine, which could not be observed in the analysis of the differences in the proportion of seroconversion and the geometric means of the YF antibody titers. The results obtained from the subsample of adults with dengue μFRNT and Zika PRNT_90_ tests did not differ substantially from those that were generated for the total adults with the rapid test (IgG). This showed consistent results despite having a smaller size and a non-probabilistic subsample, but with testing based on neutralizing antibodies that had greater accuracy. These results indicated that the interference of previous orthoflavivirus infections in the level of immunity for YF may add to the need for booster doses. We also recognize that other covariates (vaccine batch and batch expiry date for example) of theoretical relevance could improve the estimates of the statistical models and partly explain the variations in the response to vaccination. Residual confounding and selection bias could also explain the increased likelihood of higher YF antibody titers in older adults, for which we know of no biologic reason.

Considering the results presented and discussed above, we concluded that the follow-up of cohort of children and adults living in an YF nonendemic area with high seropositivity rates for other orthoflaviruses added more robust evidence compared to that from previous cross-sectional studies in endemic areas, sometimes without data on the exact moment of vaccination and with scarce data on dengue and Zika serological status. Our data strengthened the recommendation of the Brazilian National Immunization Program for a booster dose of the vaccine at 4 years of age. Current recommendations based on the assumption of life-long protection of a single dose of the YF vaccine do not seem to sustain the level of protection required in high-risk areas, especially where infants are the preferred target for vaccination. Follow-up of our cohort will show whether and when adults should get a booster dose.

## Supporting information

S1 Fig Cutoff points obtained by segmented models of the outcome neutralizing antibody titer for yellow fever 30–45 days after vaccination. These segmented models were adjusted using segmented of a generalized linear model with a Gamma distribution (outcome: YF antibody titer 30–45 days; explanatory variable: key participant index). A-sample of children who had IgG serology for dengue and Zika. B-sample of adults who had IgG serology for dengue and Zika. C-subsample of children with neutralizing antibody titers for Zika and dengue. D-subsample of adults with neutralizing antibody titers for Zika and dengue.
(TIF)

S1 Table Absolute and relative frequencies of seropositivity pre-vaccine, 30–45 days and 1 year after yellow fever vaccination according to age range, in the municipalities of Alhandra, Conde and Caaporã. (DOCX)

S2 Table Distribution of participants by serological status for yellow fever 30–45 days after vaccination, stratified by IgG serology for dengue and Zika pre-vaccine and 30–45 days after vaccination. (DOCX)

S3 Table Distribution of participants by serological status for yellow fever 1 year after vaccination, stratified by IgG serology for dengue and Zika pre-vaccine and 30–45 days and 1 year after vaccination. (DOCX)

S4 Table Geometric means and 95% confidence intervals of yellow fever neutralizing antibody titers (µFRNT; reciprocal of the dilution) in children according to serological status (neutralizing antibodies) for dengue (µFRNT) and Zika (PRNT_90_) in 3 timepoints. (DOCX)

S5 Table Geometric means and 95% confidence intervals of yellow fever neutralizing antibody titers (µFRNT; reciprocal of the dilution) in adults according to serological status for dengue (µFRNT) and Zika (PRNT_90_) in 3 timepoints. (DOCX)

S1 Appendix Collaborative group for yellow fever vaccine studies. (DOCX)
