# Supplementary figures and images for: Immunogenicity of a single dose of the 17DD yellow fever vaccine in a cohort of adults and children in a non-endemic area, and its association with dengue and Zika seropositivity
Source: PLoS Negl Trop Dis. 2025 Apr 9;19(4):e0012993. doi: 10.1371/journal.pntd.0012993 (PMC12047785; doi:10.1371/journal.pntd.0012993)

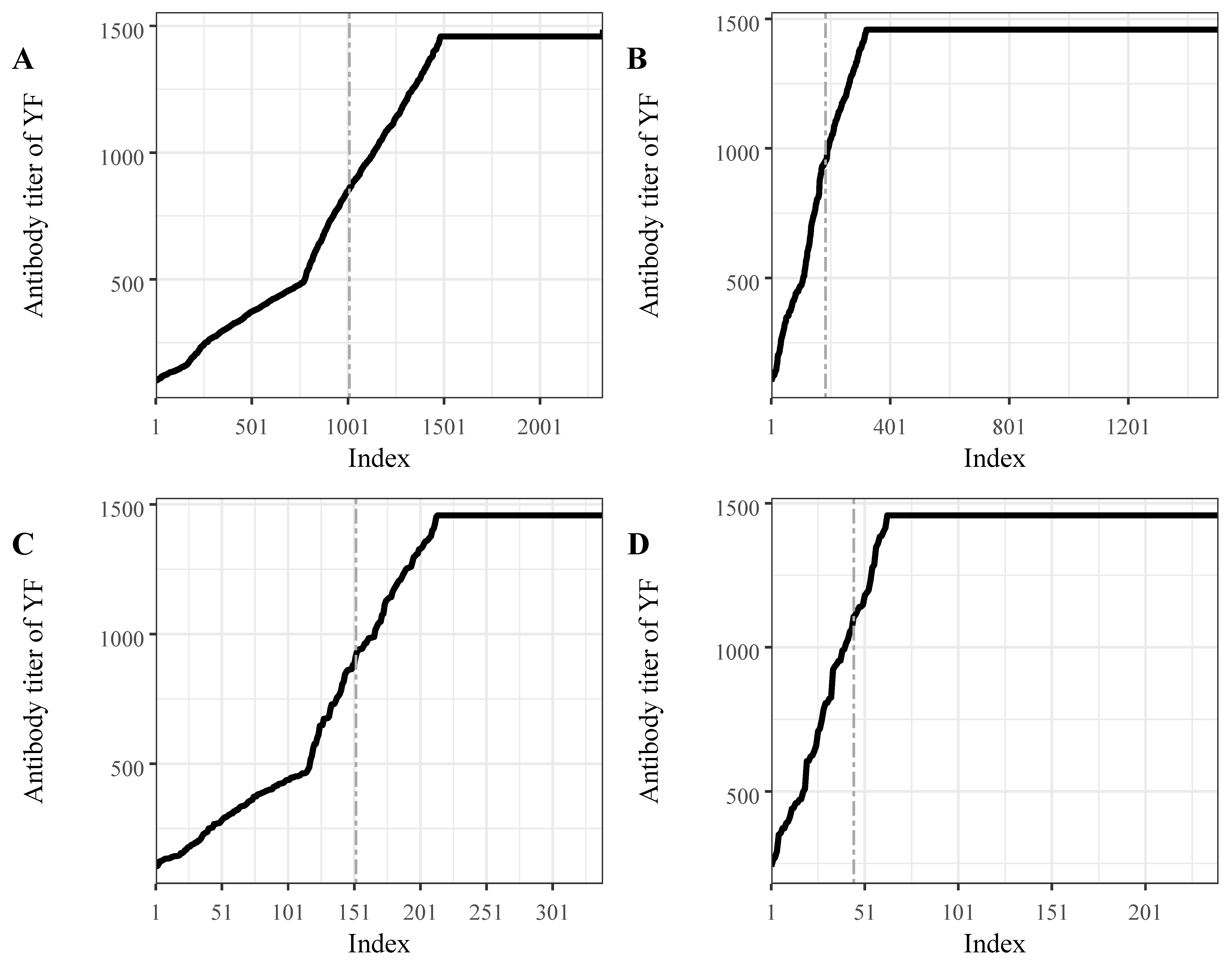

Supplement: S1 Fig — These segmented models were adjusted using segmented of a generalized linear model with a Gamma distribution (outcome: YF antibody titer 30–45 days; explanatory variable: key participant index). A-sample of children who had IgG serology for dengue and Zika. B-sample of adults who had IgG serology for dengue and Zika. C-subsample of children with neutralizing antibody titers for Zika and dengue. D-subsample of adults with neutralizing antibody titers for Zika and dengue. (TIF) [file pntd.0012993.s001.tif]
