# Supplementary material for: Immunogenicity of a single dose of the 17DD yellow fever vaccine in a cohort of adults and children in a non-endemic area, and its association with dengue and Zika seropositivity
Source: PLoS Negl Trop Dis. 2025 Apr 9;19(4):e0012993. doi: 10.1371/journal.pntd.0012993 (PMC12047785; doi:10.1371/journal.pntd.0012993)
Supplement: S1 Table — (DOCX) [file pntd.0012993.s002.docx]

| **Variables** | **Age range** | | | | | | | | |
| --- | --- | --- | --- | --- | --- | --- | --- | --- | --- |
|  | **9 to 23 months** | | | **2 to 4 years** | | **18 to 35 years** | | **36 to 50 years** | |
|  | **n** | **%** | | **n** | **%** | **n** | **%** | **n** | **%** |
| **YF seropositivity pre-vaccination** |  | | |  | |  | |  |  |
| Alhandra | 0 | | 0 | 2 | 1.1 | 31 | 8.5 | 13 | 6.4 |
| Conde | 27 | | 6.7 | 37 | 4.3 | 138 | 25.0 | 90 | 28.6 |
| Caaporã | 11 | | 2.8 | 21 | 2.4 | 80 | 22.0 | 61 | 29.5 |
| **YF seropositivity 30-45 days** |  | | |  | |  | |  |  |
| Alhandra | 50 | 92.6 | | 173 | 95.1 | 351 | 98.9 | 201 | 99.5 |
| Conde | 365 | 91.5 | | 810 | 94.2 | 542 | 98.9 | 312 | 99.0 |
| Caaporã | 350 | 90.9 | | 825 | 96.3 | 355 | 98.9 | 208 | 100.0 |
| **YF seropositivity**  **1 year** |  |  | |  | |  | |  |  |
| Alhandra | 44 | 86.3 | | 153 | 90.5 | 332 | 96.8 | 193 | 99.0 |
| Conde | 350 | 89.7 | | 771 | 91.0 | 525 | 97.8 | 304 | 97.7 |
| Caaporã | 328 | 87.2 | | 787 | 93.4 | 344 | 99.1 | 206 | 100.0 |
