# Supplementary material for: Immunogenicity of a single dose of the 17DD yellow fever vaccine in a cohort of adults and children in a non-endemic area, and its association with dengue and Zika seropositivity
Source: PLoS Negl Trop Dis. 2025 Apr 9;19(4):e0012993. doi: 10.1371/journal.pntd.0012993 (PMC12047785; doi:10.1371/journal.pntd.0012993)
Supplement: S2 Table — (DOCX) [file pntd.0012993.s003.docx]

| **Variables** | **μFRNT yellow fever 30-45 days after vaccination** | | | | | | **p-value** |
| --- | --- | --- | --- | --- | --- | --- | --- |
|  | **Seronegative** | | **Indeterminate** | | **Seropositive** | |  |
|  | **n** | **%** | **n** | **%** | **n** | **%** |  |
| **IgG dengue pre-vaccination** |  |  |  | |  |  | <0.001 |
| Negative | 95 | 3.9 | 38 | 1.6 | 2,281 | 94.5 |  |
| Positive | 19 | 1.2 | 8 | 0.5 | 1,580 | 98.3 |  |
| **IgG dengue 30-45 days** |  |  |  | |  |  | <0.001 |
| Negative | 90 | 4.1 | 39 | 1.8 | 2,070 | 94.1 |  |
| Positive | 24 | 1.2 | 13 | 0.7 | 1,901 | 98.1 |  |
| **IgG Zika pre-vaccination** |  |  |  | |  |  | <0.001 |
| Negative | 99 | 3.5 | 42 | 1.5 | 2,718 | 95.0 |  |
| Positive | 17 | 1.3 | 12 | 0.9 | 1,294 | 97.8 |  |
| **IgG Zika 30-45 days** |  |  |  | |  |  | <0.001 |
| Negative | 101 | 3.6 | 43 | 1.6 | 2,639 | 94.8 |  |
| Positive | 15 | 1.1 | 11 | 0.8 | 1,360 | 98.1 |  |

Pre-vaccine yellow fever seropositive individuals excluded.
