# Supplementary material for: Immunogenicity of a single dose of the 17DD yellow fever vaccine in a cohort of adults and children in a non-endemic area, and its association with dengue and Zika seropositivity
Source: PLoS Negl Trop Dis. 2025 Apr 9;19(4):e0012993. doi: 10.1371/journal.pntd.0012993 (PMC12047785; doi:10.1371/journal.pntd.0012993)
Supplement: S3 Table — (DOCX) [file pntd.0012993.s004.docx]

| **Variables** | **μFRNT yellow fever 1 year after vaccination** | | | | | | **p-value** |
| --- | --- | --- | --- | --- | --- | --- | --- |
|  | **Seronegative** | | **Indeterminate** | | **Seropositive** | |  |
|  | **n** | **%** | **n** | **%** | **n** | **%** |  |
| **IgG dengue 30-45 days** |  |  |  | |  |  | <0.001 |
| Negative | 117 | 5.5 | 47 | 2.2 | 1,971 | 92.3 |  |
| Positive | 69 | 3.6 | 16 | 0.8 | 1,814 | 95.6 |  |
| **IgG dengue 1 year** |  |  |  | |  |  | <0.001 |
| Negative | 123 | 6.2 | 41 | 2.1 | 1,819 | 91.7 |  |
| Positive | 64 | 3.0 | 20 | 1.0 | 1,998 | 96.0 |  |
| **IgG Zika 30-45 days** |  |  |  | |  |  | <0.001 |
| Negative | 140 | 5.2 | 52 | 1.9 | 2,512 | 92.9 |  |
| Positive | 50 | 3.7 | 10 | 0.7 | 1,300 | 95.6 |  |
| **IgG Zika 1 year** |  |  |  | |  |  | <0.001 |
| Negative | 163 | 5.3 | 55 | 1.8 | 2,874 | 92.9 |  |
| Positive | 27 | 2.7 | 7 | 0.7 | 963 | 96.6 |  |

Pre-vaccine yellow fever seropositive individuals excluded.
