# Supplementary material for: Immunogenicity of a single dose of the 17DD yellow fever vaccine in a cohort of adults and children in a non-endemic area, and its association with dengue and Zika seropositivity
Source: PLoS Negl Trop Dis. 2025 Apr 9;19(4):e0012993. doi: 10.1371/journal.pntd.0012993 (PMC12047785; doi:10.1371/journal.pntd.0012993)
Supplement: S4 Table — (DOCX) [file pntd.0012993.s005.docx]

| **Subgroups** | | **Geometric mean (95% CI) of yellow fever antibody titers** | | |
| --- | --- | --- | --- | --- |
|  |  | **pre-vaccination** | **30-45 days post-vaccination** | **1 year post-vaccination** |
| **μFRNT dengue pre-vaccination** | seropositive | **34 (31-37)** | **537 (455-634)** | - |
|  | seronegative | **31 (28-34)** | **700 (613-798)** | - |
| **PRNT_90_ Zika pre-vaccination** | seropositive | 32 (29-35) | 624 (534 – 729) | - |
|  | seronegative | 32 (29-35) | 609 (526-704) | - |
| **μFRNT dengue and PRNT_90_ Zika pre-vaccination** | seropositive | 35 (32-38) | 573 (477-688) | - |
|  | At least 1 negative | **39 (33-45)** | 596 (465-763) | - |
|  | seronegative | 32 (29-36) | 665 (571-775) | - |
| **μFRNT dengue 30-45 days** | seropositive | - | 646 (556-751) | **243 (205-288)** |
|  | seronegative | - | 582 (490-690) | **347 (291-413)** |
| **PRNT_90_ Zika 30-45 days** | seropositive | - | 666 (569-779) | 260 (219-308) |
|  | seronegative | - | 578 (492-679) | 304 (255-364) |
| **μFRNT dengue and PRNT_90_ Zika 30-45 days** | seropositive | - | 683 (582-800) | 262 (220-313) |
|  | At least 1 negative | - | 515 (363-731) | **193 (130-286)** |
|  | seronegative | - | 593 (498-706) | **357 (298-427)** |

Pre-vaccine yellow fever seropositive individuals excluded.

Data from the subsample of participants tested for μFRNT dengue and PRNT_90_ Zika.

Bold: emphasis on statistically significant differences between categories of variables.
