# Supplementary material for: Immunogenicity of a single dose of the 17DD yellow fever vaccine in a cohort of adults and children in a non-endemic area, and its association with dengue and Zika seropositivity
Source: PLoS Negl Trop Dis. 2025 Apr 9;19(4):e0012993. doi: 10.1371/journal.pntd.0012993 (PMC12047785; doi:10.1371/journal.pntd.0012993)
Supplement: S5 Table — (DOCX) [file pntd.0012993.s006.docx]

| **Subgroups** | | **Geometric mean (95% CI) of yellow fever antibody titers** | | |
| --- | --- | --- | --- | --- |
|  |  | **pre-vaccination** | **30-45 days post-vaccination** | **1 year post-vaccination** |
| **μFRNT dengue pre-vaccination** | seropositive | 35 (32-39) | **1,126 (1,034-1,227)** | - |
|  | seronegative | 34 (29-39) | **1,355 (1,284-1,430)** | - |
| **PRNT_90_ Zika pre-vaccination** | seropositive | **55 (46-66)** | 1,232 (1,028-1,477) | - |
|  | seronegative | **31 (28-34)** | 1,196 (1,122-1,275) | - |
| **μFRNT dengue and PRNT_90_ Zika pre-vaccination** | seropositive | **56 (46-67)** | 1,211 (992-1,479) | - |
|  | At least 1 negative | 36 (33-40) | 1,119 (1,019-1,228) | - |
|  | seronegative | 34 (29-39) | 1,352 (1,280-1,429) | - |
| **μFRNT dengue 30-45 days** | seropositive | - | **1,076 (965-1,200)** | **726 (641-821)** |
|  | seronegative | - | **1,290 (1,185-1,405)** | **1,006 (867-1,167)** |
| **PRNT_90_ Zika 30-45 days** | seropositive | - | **1,302 (1,154-1,469)** | 784 (579-1,061) |
|  | seronegative | - | **1,107 (1,008-1,216)** | 791 (710-881) |
| **μFRNT dengue and PRNT_90_ Zika 30-45 days** | seropositive | - | 1,302 (1,154-1,469) | 784 (579-1,061) |
|  | At least 1 negative | - | **1,038 (914-1,179)** | 715 (624-820) |
|  | seronegative | - | 1,290 (1,185-1,405) | 1,006 (867-1,167) |

Pre-vaccine yellow fever seropositive individuals excluded.

Data from the subsample of participants tested for μFRNT dengue and PRNT_90_ Zika.

Bold: emphasis on statistically significant differences between categories of variables.
