# Supplementary material for: Immunogenicity of a single dose of the 17DD yellow fever vaccine in a cohort of adults and children in a non-endemic area, and its association with dengue and Zika seropositivity
Source: PLoS Negl Trop Dis. 2025 Apr 9;19(4):e0012993. doi: 10.1371/journal.pntd.0012993 (PMC12047785; doi:10.1371/journal.pntd.0012993)
Supplement: S1 Appendix — (DOCX) [file pntd.0012993.s007.docx]

**S1 Appendix**

**Collaborative Group for Yellow Fever Vaccine Studies**. *Steering Committee*: Maria de Lourdes de Sousa Maia, Luiz Antonio Bastos Camacho, Eduardo Sérgio Soares Sousa (Principal Investigator). *Collaborating centers*: Departamento de Assuntos Médicos (DEAME), Bio-Manguinhos, Fiocruz (coordinating center of all study activities; clerical work and site of data management and analysis); Centro de Ciências Médicas, Universidade Federal da Paraíba (site of coordination of field work), Hospital Universitário Lauro Wanderley, Universidade Federal da Paraíba (health care in case of severe adverse events and management of blood samples); Laboratório Central de Saúde Pública, state of Paraíba (early processing of blood samples and storage of serum aliquots); Health Secretary, state of Paraíba (storage and distribution of the yellow fever vaccine to the municipalities); Health Secretaries at the municipalities of Alhandra, Caaporã and Conde (provided rooms in health units to install facilities for participants´ interviews, vaccination, blood collection and assistance of adverse events; supported activities of field work, including recruitment of participants and return of blood test results; and meetings with health workers to present study results); Laboratório de Análise Imunomolecular, Bio-Manguinhos, Fiocruz (performed titration of neutralizing antibodies for yellow fever and dengue); Laboratório de Arbovírus e Vírus Hemorrágicos (LARBOH) – Instituto Oswaldo Cruz/Fiocruz (performed chemiluminiscence tests for dengue and Zika); Instituto René Rachou-FIOCRUZ/Minas (performed analyses of cellular immunity).
